# The Nuances of Cost-Related Medication Non-Adherence in Patients Receiving Immunosuppression Compared to Patients with Chronic Disease: A Cross-Sectional Study

**DOI:** 10.3390/jpm16100524

**Published:** 2026-10-10

**Authors:** Samuel D. Good, Bryant R. England, Elizabeth R. Volkmann

**Affiliations:** 1 Division of Rheumatology, University of California Los Angeles, Los Angeles, CA 90230, USA; 2 Division of Rheumatology and Immunology, University of Nebraska Medical Center, Omaha, NE 68198, USA; 3 Rheumatology, Veterans Affairs Nebraska-Western Iowa Health Care System, Omaha, NE 68105, USA

**Keywords:** medication adherence, immunosuppression, personalized medicine

## Abstract

**Background**: Medication adherence rates across diseases that require immunosuppression, including autoimmune diseases, are low. While cost barriers affect medication adherence, it is unknown whether adherence rates related to cost barriers differ between medically immunosuppressed patients and similar patients with other chronic diseases. The purpose of this study is to evaluate the influence of immunosuppression on cost-related medication non-adherence (CRMNA). **Methods**: We performed a cross-sectional study utilizing the National Health Interview Survey (NHIS). Multivariable logistic regression was used to evaluate the association between immunosuppression and CRMNA, accounting for sex, age, education, race, income, life satisfaction, insurance status and marital status. Predicted probabilities of CRMNA were estimated by age group, and a subgroup analysis was performed among people who described as having poor health. **Results**: In our unweighted sample of 17,394 adults, 54.3% were female, 12.8% self-identified as Black, 35.2% were publicly insured (Medicare and/or Medicaid), and 11.1% were below the federal poverty level. The prevalence of reporting CRMNA was 11% for patients with chronic diseases and no immunosuppression, 12.5% for patients with immunosuppression but no chronic disease, and 19.1% for patients with chronic disease and immunosuppression. In the multivariable model, participants with medical immunosuppression and chronic diseases were more likely to report CRMNA than participants with chronic diseases but no immunosuppression (OR 1.55, 95% CI [1.24, 1.94], *p* = 0.001). Participants with medical immunosuppression but no chronic disease did not differ significantly from the group with chronic diseases and no immunosuppression (OR 0.87, 95% CI [0.60, 1.29], *p* = 0.49). The predicted probability of CRMNA was highest for 30–49-year-old immunosuppressed individuals with one or more chronic diseases at 23%. CRMNA was similarly associated with immunosuppression status in patients with poor health. **Conclusions**: Medically immunosuppressed individuals with chronic disease experience greater CRMNA than individuals with chronic disease who are not immunosuppressed, particularly among individuals 30–49 years of age. Future studies aimed at reducing CRMNA in immunosuppressed individuals may consider interventions that target the role of chronic health conditions in non-adherence.

## 1. Introduction

Medication adherence rates among individuals taking immunosuppressive medications, such as patients with rheumatic diseases, are low [1,2,3,4,5,6,7,8,9]. Rates of medication adherence in people with rheumatic diseases range from 10 to 80% depending on the underlying disease [6,10,11,12]. The evaluation of medication non-adherence in rheumatic diseases is essential, because it is well-known that non-adherence is associated with worse outcomes across the disease spectrum [5,13,14].

While multiple factors affect medication adherence, cost plays a central role in non-adherence. For example, in rheumatoid arthritis (RA), prior studies have found that for every $5.50 increase in the weekly out-of-pocket cost of etanercept or adalimumab, one week of adherence to therapy is lost [4]. Even the perception that medications are expensive leads to higher non-adherence in RA patients [14].

However, minimal data exist on medication adherence rates related to cost barriers among medically immunosuppressed patients who live with chronic diseases. It is also unknown whether medication adherence rates differ between medically immunosuppressed patients with and without chronic diseases. The purpose of this study was to evaluate how cost-related medication non-adherence (CRMNA) differs between patients receiving immunosuppression with and without chronic diseases (e.g., hypertension, diabetes mellitus, asthma). Using cross-sectional data from the National Health Interview Survey (NHIS) in 2023, our goals were to: (1) evaluate the factors associated with non-adherence in a nationally representative sample of immunosuppressed patients; and (2) determine whether the odds of reporting CRMNA differ between patients with medical immunosuppression and patients with other chronic disease. As has been shown previously [15,16,17], we hypothesized that patients with medical immunosuppression would have higher odds of reporting CRMNA than patients without medical immunosuppression due to high medical complexity and out-of-pocket costs. For similar reasons, we further hypothesized that patients with both medical immunosuppression and chronic diseases would have increased odds of reporting CRMNA.

## 2. Materials and Methods

### 2.1. Study Design and Data

We conducted a cross-sectional analysis of data collected from the NHIS database from 1 January 2023 to 31 December 2023, the most up-to-date complete sample at the time of data analysis. The NHIS is a nationally representative household interview survey targeting the civilian, non-institutionalized population residing within the United States [18]. Survey respondents were categorized into one of three groups: (1) medically immunosuppressed patients (i.e., patients receiving medications known to suppress the immune system) without other chronic health conditions; (2) patients with chronic health conditions without medical immunosuppression; and (3) medically immunosuppressed patients AND with the presence other chronic health conditions. Patient categorization of immunosuppression and chronic disease status was based on self-reporting through NHIS questionnaire responses. For medical immunosuppression status, patients were asked, “In the past 12 months, have you taken prescription medication or had any medical treatments that a doctor or other health professional told you would weaken your immune system?” (NHIS variable: MEDRXTRT_A). Responses were operationalized as a binary indicator (“yes” vs. “no”). We excluded sample adults with a response of “refused” or “don’t know.” There are limited data on the validity of self-reporting of immunosuppression status when compared to clinical diagnoses. However, previous studies have found >80% agreement between self-reported chronic disease status and physician diagnoses [19]. This study was conducted in accordance with Strengthening the Reporting of Observational Studies in Epidemiology (STROBE) guidelines.

### 2.2. Chronic Health Conditions

Chronic diseases used as a comparator group for the purposes of this study included hypertension, hypercholesterolemia, coronary artery disease, asthma, diabetes, chronic obstructive pulmonary disease (COPD), or any combination of the above diagnoses. We selected the included chronic diseases based on the fact that these are lifelong processes that require ongoing medication management similar to diseases that require immunosuppression. Furthermore, chronic heart and lung disease have been utilized in the literature previously as important comorbidities for health outcomes in immunosuppressed patients [20].

We excluded chronic kidney disease (CKD), cancer, depression, arthritis, and heart failure from the ‘chronic health conditions’ comparator group because either (1) the diseases differ substantially from our group of interest, namely medically immunosuppressed patients, or (2) their inclusion could bias our results. Arthritis, as ascertained in NHIS, explicitly includes rheumatoid arthritis and lupus and would therefore capture individuals with immunosuppression alongside patients without immunosuppression, such as fibromyalgia. Cancer and advanced CKD are also associated with treatment-related immunosuppression, which could bias estimates toward the null. Finally, heart failure and depression differ substantially from the immunosuppressed population in terms of affected demographics (different age distributions) and the duration of treatment, respectively.

### 2.3. Covariables

We included potentially confounding covariates that may affect the relationship between the primary regressor (immunosuppression status or chronic disease status) and the outcome of interest (CRMNA). Based on the existing literature on CRMNA, the covariates included in this model include sex, self-reported life satisfaction, race, age, self-reported general health status, education, insurance status, family income, and marital status [21,22,23]. Including race as a variable without careful consideration of its role in a study outcome can minimize or trivialize the impact of racism on health outcomes [24]. Acknowledging this concern, we opted to include race as a covariate because systemic racism affects how medications are prescribed by providers and received by patients [25].

### 2.4. Outcome

The primary outcome of this study was CRMNA. NHIS includes several questions that evaluate CRMNA, including whether survey respondents in the past 12 months “skipped medication doses to save money,” “took less medication to save money,” “delayed filling a prescription to save money,” and “needed prescription medication but did not get it due to cost.” A “yes” response to 1 or more of the 4 questions listed above met the criteria for CRMNA. A similar approach has been cited previously [26]. All other patients (i.e., those who said “no” to all 4 of the questions above) were defined as not having CRMNA.

### 2.5. Statistical Analysis

Descriptive statistics were used to describe survey responses prior to accounting for survey weighting and complex survey design. Since NHIS uses sampling weights and complex survey design so that the total survey is representative of the population of the United States [18], all other statistical analyses (excluding descriptive analyses) were conducted accounting for weighting and survey design.

All analyses accounted for the NHIS complex, multistage sampling design using the survey (svy) procedures in STATA SE version 18 (StataCorp LLC, College Station, TX, USA). We specified the sample adult final annual weight (WTFA_A) as the sampling weight, the pseudo-stratum variable (PSTRAT) as the stratum, and the pseudo-primary sampling unit variable (PPSU) as the primary sampling unit. Analyses used STATA’s svy procedures with Taylor series linearization for variance estimation. To address strata containing a single PSU, we used STATA’s scaled approach (single unit (scaled)), in which each singleton stratum is assigned the average variance contribution of strata containing multiple PSUs.

A multivariable logistic regression model was used to evaluate the association between immunosuppression use and CRMNA when controlling for all other covariates. For the variable selection process, all variables were included a priori based on our conceptual model of factors that were likely to affect CRMNA. Results are reported as odds ratios (ORs), 95% CIs and *p*-values. Given that wealth varies across the lifespan, predicted probabilities of CRMNA were estimated by age group. This analysis was conducted to present our results in a more easily interpretable format but is not intended to imply effect modification between age and immunosuppression status on CRMNA. Analyses were conducted using a complete-case approach. Finally, a post hoc exploratory subgroup analysis was performed among people reporting poor health to describe a particular population at high risk for developing CRMNA. All data were analyzed using STATA SE version 18 (StataCorp LLC, College Station, TX, USA).

## 3. Results

In our unweighted sample of 17,394 adults, 54.3% were female, 12.8% self-identified as Black, 35.2% were publicly insured, and 11.1% reported an income below the federal poverty level (Supplementary Table S1, reported as unweighted observations for clarity). The majority of patients in our sample were not medically immunosuppressed (N = 15,866; 91.2%). Among the patients who were medically immunosuppressed (N = 1528; 8.8%), few patients reported immunosuppression without concomitant chronic diseases (N = 450; 2.6%). Of the 17,394 sample adults, missing data were minimal for most covariates: sex (N = 6; <0.1%), self-reported general health (N = 20; 0.1%), age (N = 65; 0.4%), life satisfaction (N = 77; 0.4%), education (N = 144; 0.8%), and insurance status (N = 173; 1.0%). Missingness was higher for marital status (N = 1288; 7.4%) and race (N = 1597; 9.2%). However, missingness was low overall, and missing values primarily reflected item nonresponse arising from the survey administration process—for example, responses coded as “refused” or “don’t know”—which we would not expect to be related to respondents’ immunosuppression status. We therefore considered the data plausibly missing completely at random.

After incorporating survey weighting, the study’s sample size comprised 139,514,573 individuals. Compared with individuals who did not experience CRMNA (89% of weighted sample), individuals who did experience CRMNA (11% of weighted sample) were more likely to be female, Black or African American, unmarried, aged 30–49 years, and less educated. The overall prevalence of reporting CRMNA in our populations of interest was 11% for patients with chronic disease but no immunosuppression, 12.5% for patients with immunosuppression but no chronic disease, and 19.1% for patients with chronic disease and immunosuppression. In addition, individuals who experienced CRMNA had lower incomes, lower life satisfaction, and were more likely to be underinsured and in fair-to-poor health (see Table 1).

**Figure 1:**
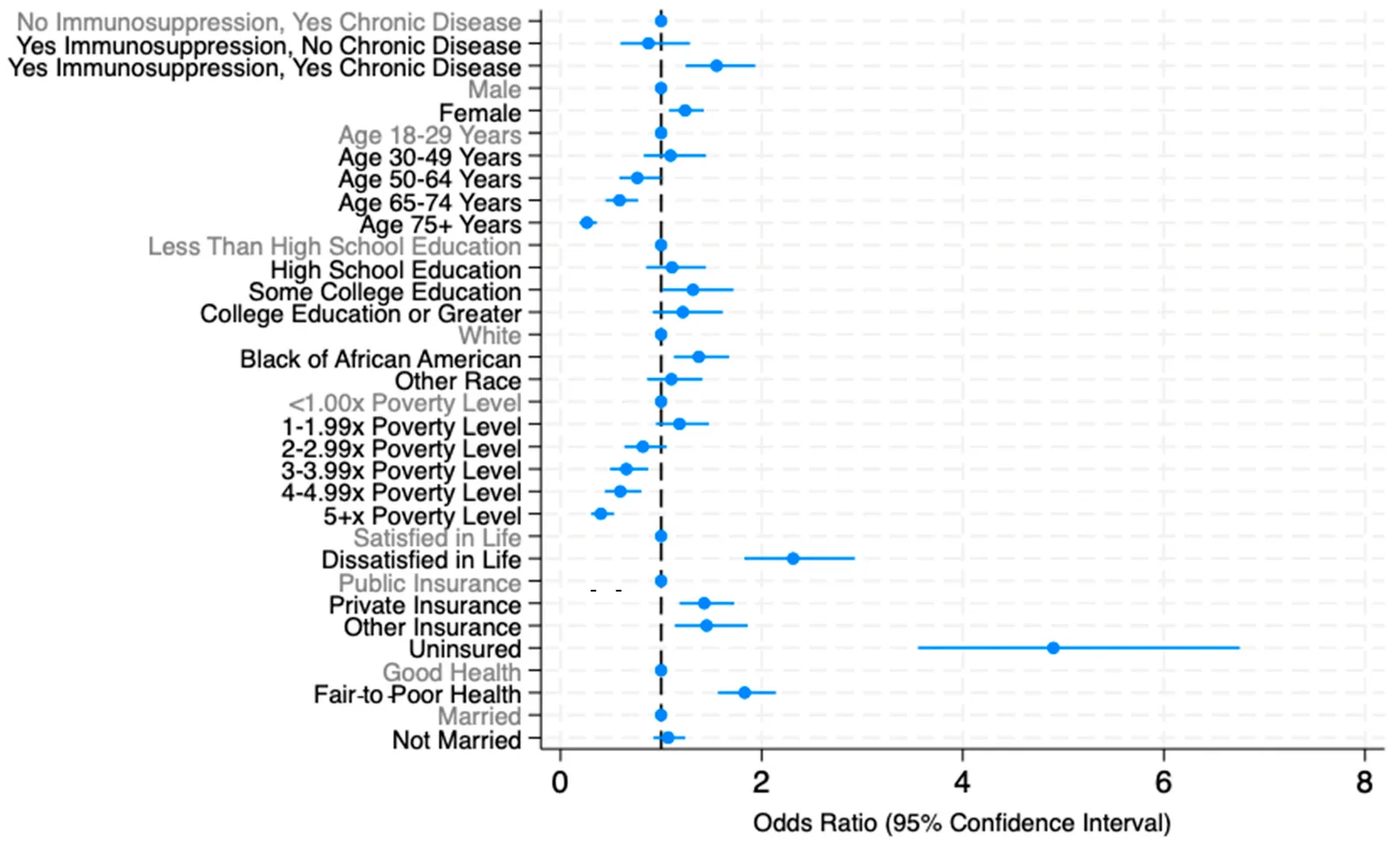
Forest plot of individual covariates’ effects on CRMNA in the final multivariable analysis (reference groups in gray).

In our final multivariable logistic regression model (Figure 1), after adjusting for all other covariates listed above, participants with medical immunosuppression and chronic diseases had 1.55-fold higher odds of reporting CRMNA compared to individuals with chronic disease and no immunosuppression (OR 1.55, 95% CI [1.24, 1.94], *p* = 0.001). There was no significant difference between individuals on medical immunosuppression alone compared to individuals with chronic diseases and no medical immunosuppression (OR 0.87, 95% CI [0.60, 1.29], *p* = 0.49). Other covariates that were associated with a greater odds of reporting CRMNA are reported in Figure 1 and Table 2, and these notably include being uninsured (OR 4.90 compared to publicly insured, 95% CI [3.56, 6.76], *p* < 0.001) and dissatisfaction in life (OR 2.31, 95% CI [1.83, 2.93], *p* < 0.001).

**Figure 2:**
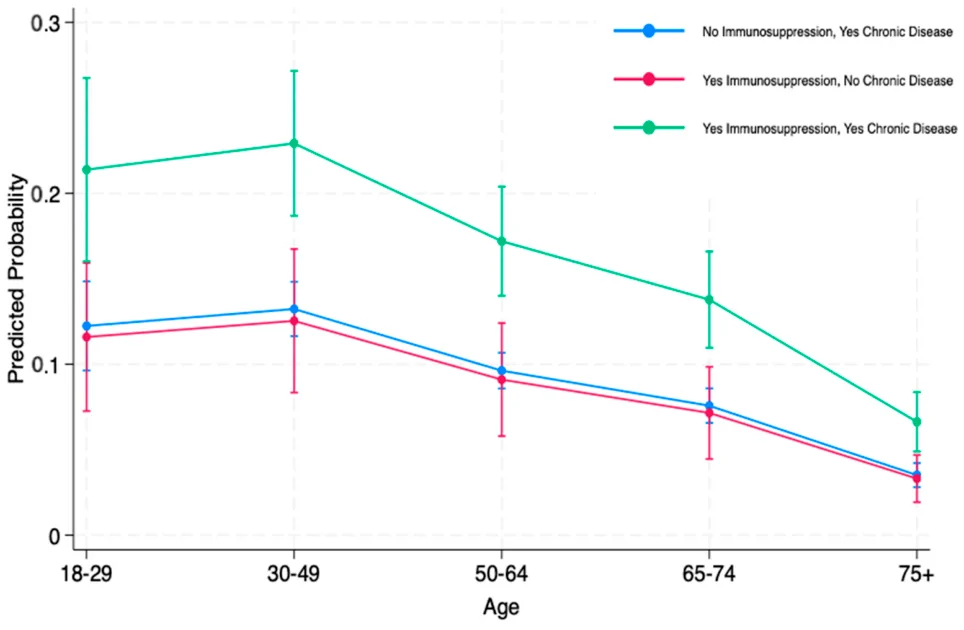
Predicted probabilities of CRMNA by age and immunosuppression subgroups.

The predicted probabilities of reporting CRMNA by age and immunosuppression status are reported in Figure 2. The predicted probability of a 30–49-year-old immunosuppressed individual with an additional chronic disease reporting CRMNA was the highest at 23% (*p* = 0.001 compared to individuals with chronic disease and no immunosuppression; *p* = 0.007 compared to individuals with immunosuppression and no chronic disease). Conversely, the predicted probability of a 75-year-old immunosuppressed individual without any chronic diseases reporting CRMNA was the lowest at 3.3% (*p* = 0.001 compared to individuals with chronic disease and no immunosuppression; *p* = 0.007 compared to individuals with immunosuppression and no chronic disease).

**Figure 3:**
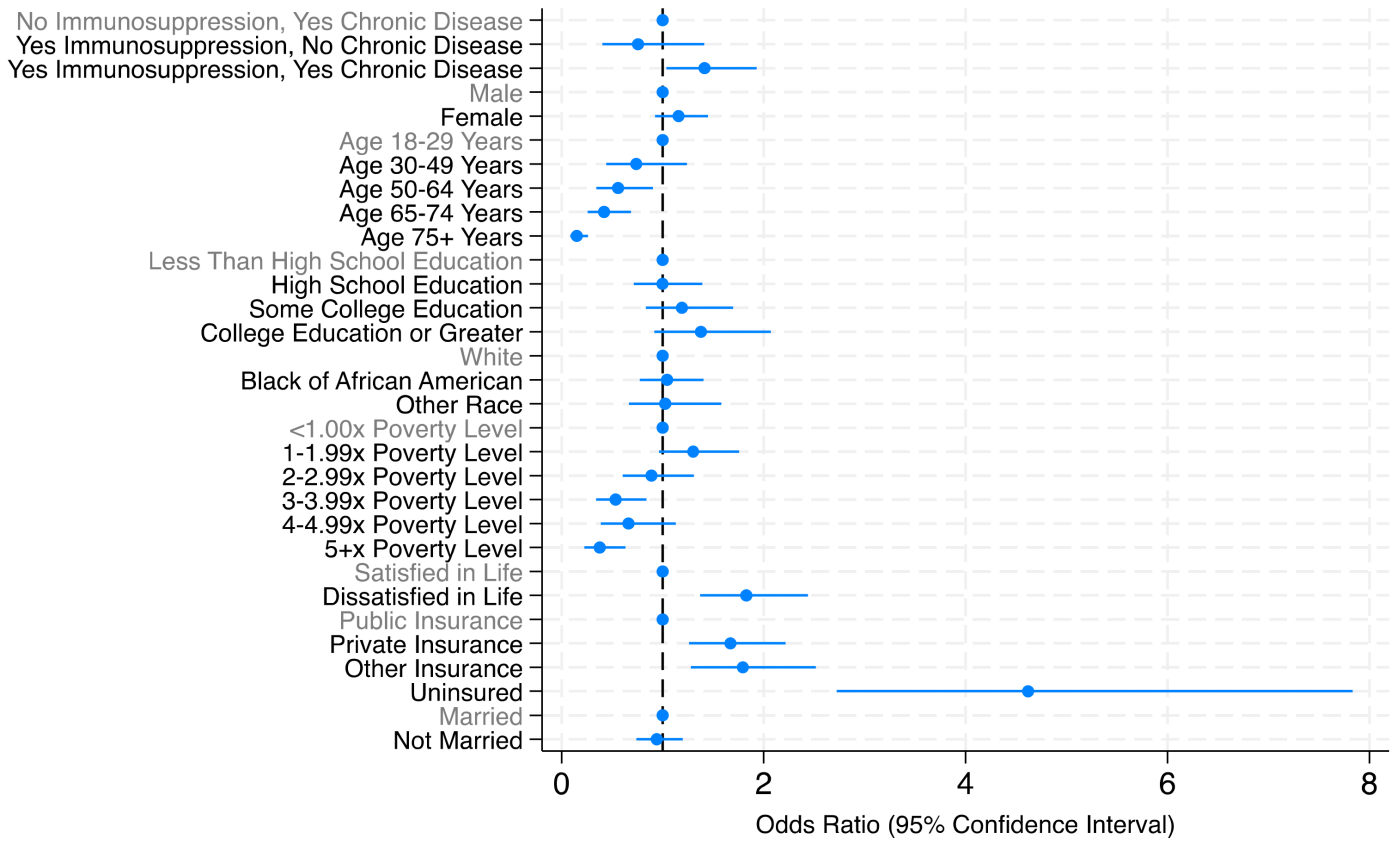
Forest plot of individual covariates’ effects on CRMNA in the subgroup multivariable analysis restricted to individuals with poor health (reference groups in gray).

A subgroup analysis was performed among individuals reporting poor health (see Figure 3) based on the results of our original multivariable analysis, which showed high odds of reporting CRMNA with relatively narrow confidence intervals in this group. Our analysis showed that CRMNA was similarly associated with immunosuppression in patients with chronic diseases (OR 1.41, 95% CI [1.04, 1.93], *p* = 0.029) but not with immunosuppression alone (OR 0.76, 95% CI [0.41, 1.41], *p* = 0.381).

## 4. Discussion

CRMNA is complicated and varies depending on factors such as age, insurance status, income, and even life satisfaction based on our findings. To our knowledge, this is the first study to explicitly model the impact of immunosuppression status on CRMNA in a nationally representative sample. Our sample size is consistent with prior studies that estimated the prevalence of chronic disease in the US non-institutionalized population [27]. The present study found that medically immunosuppressed patients with chronic health conditions experienced greater odds of reporting CRMNA than patients with chronic health conditions who were not immunosuppressed when controlling for patient demographics. However, medical immunosuppression alone did not significantly affect the odds of reporting CRMNA. Furthermore, the predicted probability of CRMNA varied significantly depending on age and immunosuppression status, ranging 3.3% to 23%. This study highlights the need for a nuanced approach to evaluating and managing immunosuppressed patients at-risk for CRMNA.

Interestingly, immunosuppression without additional medical comorbidities did not affect the odds of reporting CRMNA, but when combined with other comorbidities, it increased the odds of reporting CRMNA. This is consistent with prior studies that have identified comorbidities as a key factor in medication non-adherence [28,29]. However, the lack of association between medication adherence and immunosuppression alone was an unexpected finding. The fear of side effects has been identified in the literature as a driver of general medication non-adherence in immunosuppressed patients, as immunosuppressive medications tend to have unfavorable side-effect profiles [30,31]. Additionally, we anticipated that the high cost of immunosuppressive medications would drive CRMNA in this population. We also identified that Black or African American individuals were associated with CRMNA. Importantly, this finding is driven by structural inequities, differences in access to care, and other social determinants of health and is not an inherent characteristic associated with race, as has been shown in previous studies [32,33].

Prior studies evaluating the role of cost in medication non-adherence in rheumatic diseases have consistently identified higher medication prices as a predictor of non-adherence [4,8,9,15,34,35,36]. We hypothesize that this relationship was mitigated by our model when controlling for income and insurance status, which may be the actual drivers of CRMNA in immunosuppressed patients. This may indicate that the burden of medication costs in patients with multiple chronic diseases is exacerbated by the addition of high-cost immunosuppressives, although immunosuppressives do not intrinsically lead to CRMNA.

The predicted probabilities of CRMNA based on age and immunosuppression status further highlight its complexity. An immunosuppressed 30–49 year old individual with additional chronic conditions had the highest overall predicted probability of CRMNA. As age increased beyond 30–49 years, the predicted probability of CRMNA in immunosuppressed and non-immunosuppressed individuals declined. This pattern of non-adherence has been shown previously, although data on the role of age in medication non-adherence is sparse. One prior study evaluating non-adherence in lung transplant recipients similarly found that a younger age is associated with non-adherence, whereas older age is protective against non-adherence [37]. Studies that evaluated the role of age in non-adherence in alternative health conditions, including trauma patients and patients on statin medications, found different patterns of non-adherence based on age unique to those disease processes [38,39]. These studies did not specifically evaluate the effect of age on cost-related non-adherence, but they demonstrate the subtle differences between factors associated with non-adherence in various disease processes.

The results of our subgroup analysis of patients with fair-to-poor overall health also emphasizes the nuanced approach needed for evaluating CRMNA. Similar to our findings from the larger population of study, while immunosuppression status alone did not affect the odds of reporting CRMNA, it did so in combination with other chronic health conditions. These exploratory results corroborate the findings of our principal results and warrant further investigation into how CRMNA differs between specific subpopulations. Prior studies have identified that overall health and health-related quality of life are associated with general non-adherence [40,41,42].

Our study has notable limitations. Given the cross-sectional study design, we were unable to report any causative link between the factors identified in our study and CRMNA. Further prospective and qualitative analyses are needed to identify causation. Second, we are unable to differentiate patients with rheumatic diseases from patients with other forms of medical immunosuppression based on the questions asked in NHIS. There are few studies that use large, nationally representative databases to evaluate rheumatic diseases, partly because rheumatic diseases are rare; as such, we believe our methodology is underutilized in rheumatology. Likewise, we are unable to differentiate non-adherence to immunosuppressive medications from other medication classes based on this dataset. We hope that this study will encourage future nationally representative questionnaires like NHIS or the National Health and Nutrition Examination Survey (NHANES) to consider including questions specific to rheumatic diseases and medication classes. Finally, all diagnoses in this study were based on patient reports. We were unable to confirm diagnoses because NHIS does not report clinical information. Furthermore, medication regimens were also based on self-reporting, and as such, we were unable to confirm immunosuppression regimens or which specific medications contributed to CRMNA. However, self-reporting medications and medication adherence tends to lead to overestimation of adherence and therefore likely biases our results against type I error [43]. As this study relied on self-reported data, we were also unable to confirm patient disease activity, duration, or medication regimen. Future prospective studies should seek to identify underlying diagnoses based on clinical data when available.

This study represents a novel approach to describing possible factors associated with CRMNA in a medically complex, nationally representative population. Understanding medication non-adherence is essential to public health because adherence is routinely associated with better outcomes in chronic diseases [44,45,46]. Despite these facts, there is scant research on the nuances of CRMNA in these populations. Our results show that medical immunosuppression, when in combination with other chronic diseases, is associated with higher odds of reporting CRMNA. The prevalence of reporting CRMNA was high in all three groups in our study, despite being a highly insured population. This may be due to persistently high out-of-pocket costs, even for adequately insured patients with both private and public insurance [47,48]. A major strength of our data was that it drew from a nationally representative database and were therefore applicable to a diverse patient population residing in the US. Furthermore, we report predicted probabilities of CRMNA, a highly applicable way to describe CRMNA to patients and apply our findings in a clinical setting. As such, the data reported in our study should encourage conversations between providers and this patient population to help identify patients who are highly likely to experience CRMNA.

## 5. Conclusions

In this study, we identified that immunosuppression in combination with other comorbidities acts as a compounded risk factor for CRMNA. This finding could reflect the role of medication regimen complexity in contributing to CRMNA, though further studies are warranted to disentangle the compounded effects of out-of-pocket costs, disease severity, and illness duration on CRMNA in this population. We also found that specific populations, especially including uninsured individuals, individuals with private insurance, those in poor health, and those with low life satisfaction, had higher odds of CRMNA. We report our findings as predicted probabilities, which should allow providers to communicate a patient’s likelihood of experiencing CRMNA in easily understandable terms. Together, these findings can help providers personalize their approach to discussing non-adherence with patients with rheumatic diseases. Importantly, our results suggest that there is no single, universal approach to addressing non-adherence; rather, conversations should be tailored to each patient’s individual risk profile.

## Figures and Tables

**Table 1:** Results of bivariate analysis of all covariates in the model, as compared by cost-related medication non-adherence (CRMNA) status (all results are reported as proportions and 95% confidence intervals; *p*-values reported as results of chi-square testing; significant *p*-values highlighted in grey).

	No CRMNA (Proportion, 95% CI)	Yes CRMNA (Proportion 95% CI)	*p*-Value
Sex			<0.001
Male	0.45 [0.4419, 0.4591]	0.40 [0.3732, 0.4226]	
Female	0.55 [0.5409, 0.5581]	0.60 [0.5774, 0.6268]	
Age			<0.001
18–29	0.148 [0.14, 0.1555]	0.158 [0.1387, 0.1786]	
30–49	0.280 [0.2718, 0.2878]	0.390 [0.3654, 0.4143]	
50–64	0.264 [0.2565, 0.2713]	0.283 [0.2615, 0.3051]	
65–74	0.176 [0.1699, 0.1819]	0.122 [0.1091, 0.1365]	
75+	0.133 [0.1279, 0.1384]	0.048 [0.04, 0.0571]	
Education			<0.001
Less than High School	0.097 [0.0905, 0.1044]	0.119 [0.1014, 0.1399]	
High School	0.259 [0.2503, 0.2669]	0.296 [0.272, 0.3221]	
Some College	0.291 [0.2832, 0.2997]	0.333 [0.3103, 0.3563]	
College or Greater	0.3528 [0.3423, 0.3636]	0.251 [0.2314, 0.2725]	
Marital Status			<0.001
Married	0.555 [0.5456, 0.5644]	0.459 [0.434, 0.4839]	
Not Married	0.445 [0.4356, 0.4544]	0.541 [0.5161, 0.566]	
Race			<0.001
White	0.798 [0.7862, 0.8098]	0.726 [0.6994, 0.7512]	
Black	0.115 [0.1064, 0.125]	0.187 [0.1651, 0.2118]	
Other	0.086 [0.0793, 0.094]	0.087 [0.073, 0.1026]	
Ratio of Family Income to Federal Poverty Level			<0.001
<1.00	0.088 [0.0817, 0.0941]	0.159 [0.1398, 0.1808]	
1–1.99	0.167 [0.1603, 0.1747]	0.271 [0.2506, 0.2932]	
2–2.99	0.165 [0.1584, 0.1712]	0.208 [0.1866, 0.2308]	
3–3.99	0.129 [0.1227, 0.1346]	0.116 [0.1007, 0.1326]	
4–4.99	0.106 [0.1009, 0.1117]	0.078 [0.0668, 0.0917]	
5+	0.346 [0.3343, 0.3569]	0.167 [0.149, 0.1877]	
General Health Status			<0.001
Good	0.824 [0.817, 0.8309]	0.669 [0.6469, 0.6904]	
Fair to Poor	0.176 [0.1691, 0.183]	0.331 [0.3096, 0.3531]	
Life Satisfaction			<0.001
Satisfied	0.96 [0.9567, 0.9633]	0.871 [0.8525, 0.8871]	
Dissatisfied	0.040 [0.0367, 0.0433]	0.129 [0.1129, 0.1475]	
Insurance Type			<0.001
Public	0.291 [0.2819, 0.301]	0.265 [0.2433, 0.288]	
Private	0.616 [0.605, 0.6263]	0.505 [0.4805, 0.5304]	
Other	0.060 [0.0554, 0.0644]	0.065 [0.0543, 0.078]	
Uninsured	0.033 [0.0299, 0.0368]	0.164 [0.144, 0.187]	
Immunosuppression Status			<0.001
No Immunosuppression, Yes Chronic Disease	0.906 [0.8999, 0.9122]	0.852 [0.8308, 0.8716]	
Yes Immunosuppression, No Chronic Disease	0.030 [0.0267, 0.0343]	0.0331 [0.0245, 0.0445]	
Yes Immunosuppression, Yes Chronic Disease	0.064 [0.0586, 0.0687]	0.115 [0.0971, 0.1347]	

**Table 2:** Table of odds ratios and 95% CIs of individual covariates’ effects on CRMNA in the final multivariable analysis.

	Odds Ratio	95% Confidence Interval	*p*-Value
Yes Immunosuppression, No Chronic Disease	0.87	0.60, 1.29	0.494
Yes Immunosuppression, Yes Chronic Disease	1.55	1.24, 1.94	<0.001
Female	1.24	1.08, 1.42	0.003
Age 30–49 Years	1.09	0.83, 1.45	0.530
Age 50–64 Years	0.76	0.58, 1.00	0.048
Age 65–74 Years	0.59	0.45, 0.77	<0.001
Age 75+ Years	0.26	0.19, 0.36	<0.001
High School Education	1.11	0.85, 1.45	0.447
Some College Education	1.32	1.01, 1.72	0.043
College Education or Greater	1.22	0.92, 1.61	0.174
Black or African American	1.37	1.12, 1.68	0.002
Other Race	1.10	0.86, 1.41	0.438
1–1.99x Poverty Level	1.18	0.95, 1.48	0.136
2–2.99x Poverty Level	0.82	0.64, 1.06	0.123
3–3.99x Poverty Level	0.65	0.49, 0.87	0.004
4–4.99x Poverty Level	0.59	0.44, 0.80	0.001
5 + x Poverty Level	0.40	0.30, 0.53	<0.001
Dissatisfied in Life	2.31	1.83, 2.93	<0.001
Private Insurance	1.43	1.18, 1.73	<0.001
Other Insurance	1.45	1.13, 1.86	0.003
Uninsured	4.90	3.56, 6.76	<0.001
Fair-to-Poor Health	1.83	1.56, 2.14	<0.001
Not Married	1.07	0.92, 1.24	0.89

## Data Availability

The data that support the findings of this study are publicly available in the National Health Interview Survey at https://www.cdc.gov/nchs/nhis/index.html (accessed on 4 October 2026).

## Supplementary Materials

The following supporting information can be downloaded at: https://www.mdpi.com/article/10.3390/jpm16100524/s1, Table S1: Patient demographics by immunosuppression and chronic disease categories (all values reported as total number in unweighted sample with percentages in parentheses).
