# Supplementary material for: The Nuances of Cost-Related Medication Non-Adherence in Patients Receiving Immunosuppression Compared to Patients with Chronic Disease: A Cross-Sectional Study
Source: J Pers Med. 2026 Oct 10;16(10):524. doi: 10.3390/jpm16100524 (PMC13706589; doi:10.3390/jpm16100524)
Supplement: Supplementary file 1 [file jpm-16-00524-s001.zip › jpm-4520271-supplementary.pdf]

Supplemental Material

Table S1: Patient demographics by immunosuppression and chronic disease categories (all values reported as total number in unweighted sample with percentages in parentheses).

	No Immunosuppression, Yes Chronic Disease	Yes Immunosuppression, No Chronic Disease	Yes Immunosuppression, Yes Chronic Disease	Total
N (unweighted)	15,866 (91.2%)	450 (2.6%)	1,078 (6.2%)	17,394 (100%)
Sex				
Male	7,383 (46.5%)	152 (33.8%)	421 (39.1%)	7,956 (45.7%)
Female	8,480 (53.5%)	298 (66.2%)	657 (54.3%)	9,435 (54.3%)
Age				
18-29	1,020 (6.4%)	70 (15.6%)	49 (4.5%)	1,139 (6.6%)
30-49	3,215 (20.3%)	167 (37.1%)	218 (20.2%)	3,600 (20.7%)
50-64	4,344 (27.4%)	126 (28.0%)	345 (32.0%)	4,815 (27.7%)
65-74	4,058 (25.6%)	57 (12.7%)	272 (25.3%)	4,387 (25.3%)
75+	3,199 (20.2%)	30 (6.7%)	193 (17.9%)	3,422 (19.7%)
Education				
Less than High School	1,510 (9.6%)	16 (3.6%)	110 (10.2%)	1,636 (9.4%)
High School	4,221 (26.7%)	104 (23.2%)	270 (25.1%)	4,595 (26.5%)
Some College	4,553 (28.8%)	130 (29.0%)	309 (28.7%)	4,992 (28.8%)
College or Greater	5,511 (34.9%)	199 (44.3%)	386 (35.9%)	6,096 (35.2%)
Marital Status				
Married	7,054 (46.2%)	200 (46.4%)	483 (46.6%)	7,737 (46.3%)
Not Married	8,200 (53.8%)	231 (53.6%)	554 (53.4%)	8,985 (53.7%)
Race				
White	12,050 (79.3%)	350 (82.0%)	858 (82.8%)	13,258 (79.6%)
Black	1,998 (13.2%)	39 (9.1%)	102 (9.8%)	2,139 (12.8%)
Other	1,140 (7.5%)	38 (8.9%)	76 (7.3%)	1,254 (7.5%)
Ratio of Family Income to Federal Poverty Level				
<1.00	1,734 (10.9%)	45 (10.0%)	155 (14.4%)	1,934 (11.1%)
1-1.99	3,120 (19.7%)	69 (15.3%)	228 (21.2%)	3,417 (19.6%)
2-2.99	2,770 (17.5%)	72 (16.0%)	190 (17.6%)	3,032 (17.4%)
3-3.99	2,034 (12.8%)	51 (11.3%)	130 (12.1%)	2,215 (12.7%)
4-4.99	1,579 (10.0%)	47 (10.4%)	107 (9.9%)	1,733 (10.0%)
5+	4,629 (29.2%)	166 (36.9%)	268 (24.9%)	5,063 (29.1%)
General Health Status				
Good	12,441 (78.5%)	328 (72.9%)	581 (53.9%)	13,350 (76.8%)
Fair to Poor	3,413 (21.5%)	122 (27.1%)	497 (46.1%)	4,032 (23.2%)
Life Satisfaction				
Satisfied	15,021 (94.9%)	426 (94.9%)	951 (88.7%)	16,398 (94.5%)
Dissatisfied	805 (5.1%)	23 (5.1%)	121 (11.3%)	949 (5.5%)
Insurance Type				
Public	5,578 (35.3%)	107 (23.8%)	414 (38.4%)	6,099 (35.2%)

Private	8,454 (53.5%)	295 (65.7%)	522 (48.5%)	9,271 (53.5%)
Other	1,113 (7.0%)	30 (6.7%)	113 (10.5%)	1,256 (7.3%)
Uninsured	649 (4.1%)	17 (3.8%)	28 (2.6%)	694 (4.0%)
